# Supplementary material for: TrbA binds and locks a sliding clamp KorB to repress transcription on multi-drug resistance plasmids
Source: J Bacteriol. 2026 Aug 11;208(9):e00286-26. doi: 10.1128/jb.00286-26 (PMC13584720; doi:10.1128/jb.00286-26)
Supplement: Supplementary file 1 [file jb.00286-26-s0001.pdf]

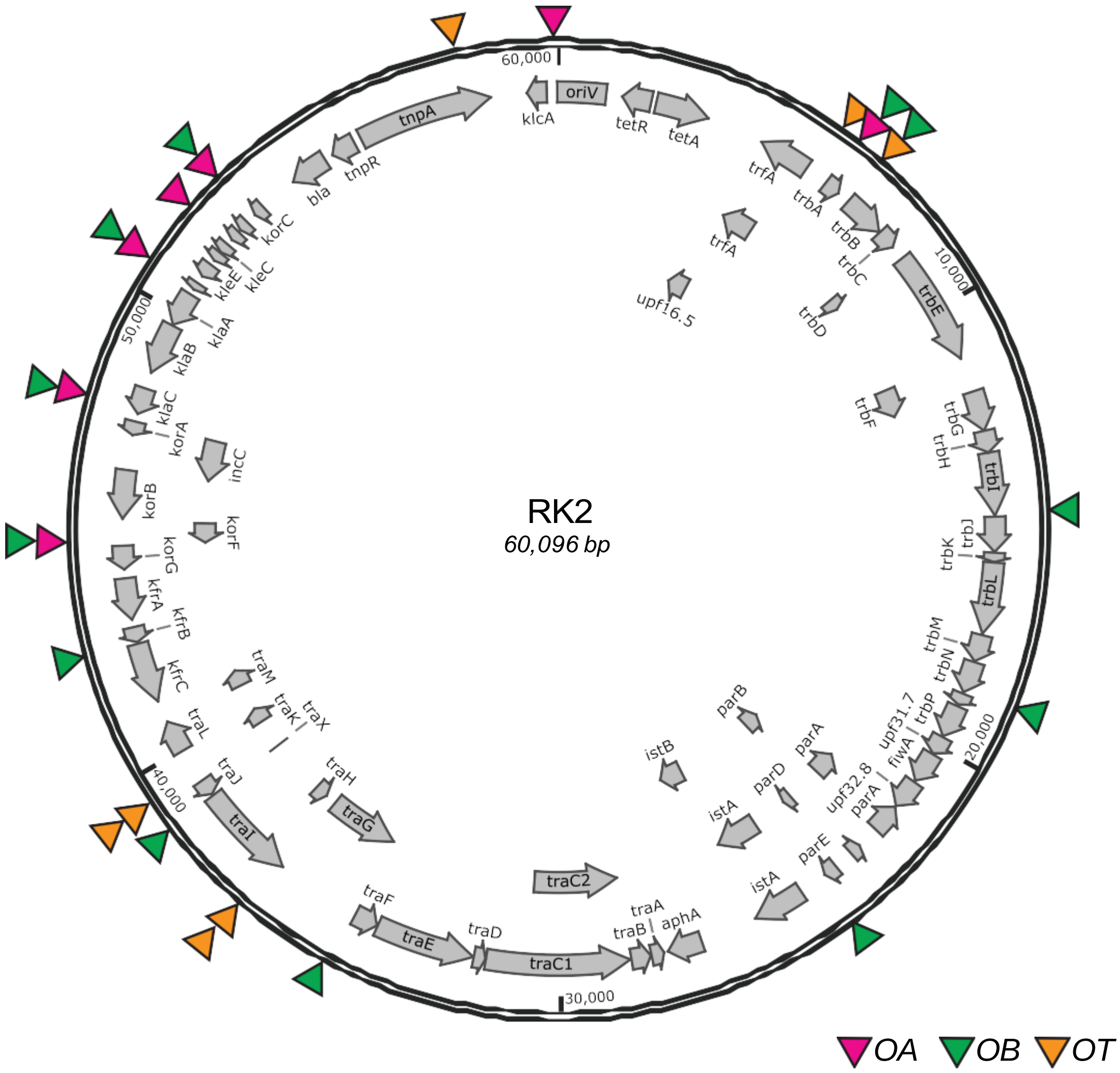

Supplementary Fig. 1. Schematic diagram of RK2 plasmid and its encoded genes. Binding sites of KorB (*OB*, green arrows), of KorA (*OA*, magenta), and of TrbA (*OT*, orange) are also shown on the RK2 plasmid map.

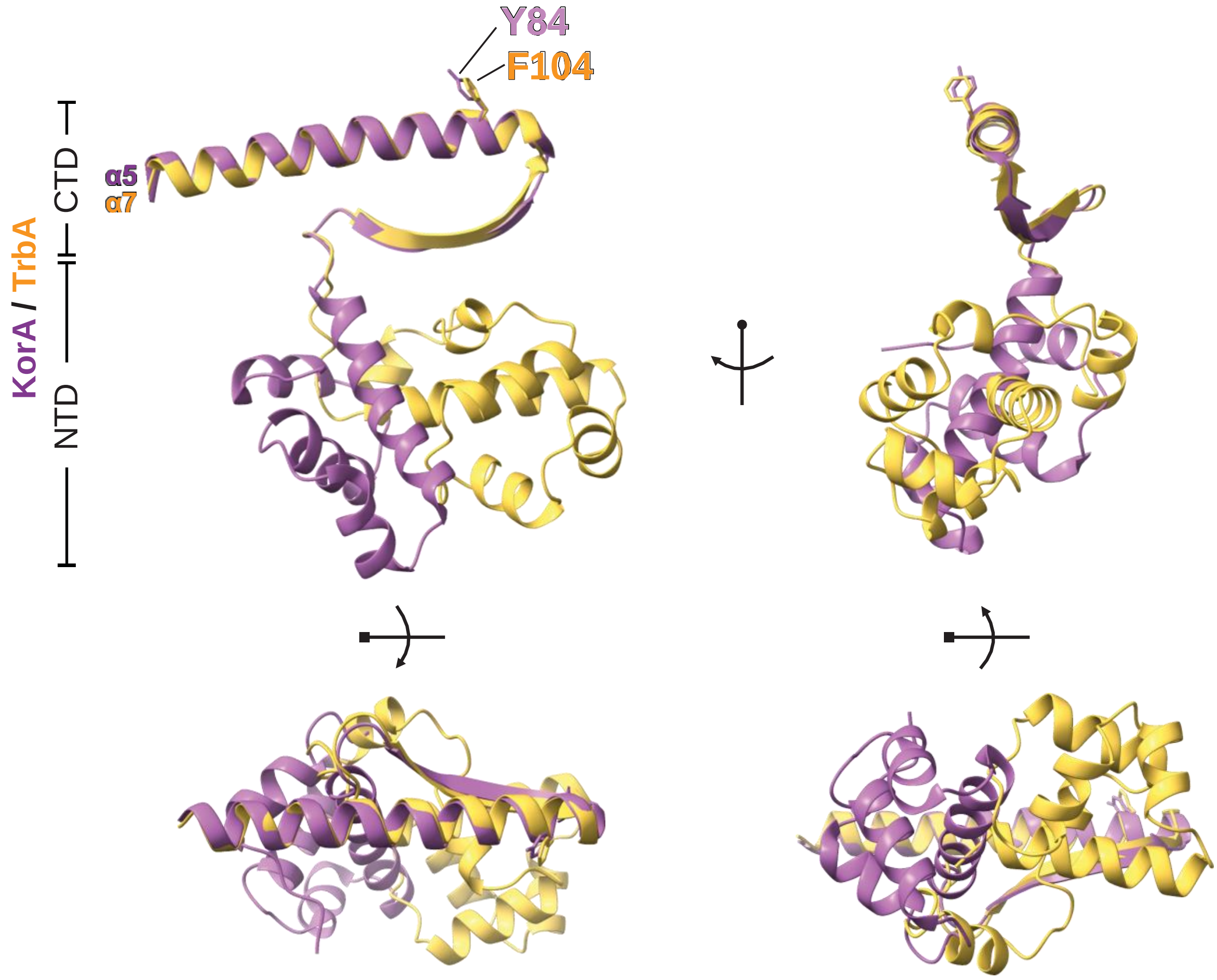

Supplementary Fig. 2. KorA and TrbA share structural homology at their C-terminal domains. Overlay of the AlphaFold2-predicted structure of TrbA monomer (yellow) on KorA monomer (purple). The C-terminal domains of KorA and TrbA closely aligned, while their N-terminal domains do not. The structure of KorA monomer is from the co-crystal structure of a KorBΔN30ΔCTD-KorA-DNA complex (PDB: 8QA9), KorBΔN30ΔCTD and the DNA were omitted for simplicity. Helix α5 (purple) of KorA and helix α7 (yellow) of TrbA, as well as the critical aromatic residue KorA Y84 and TrbA F104, are shown.

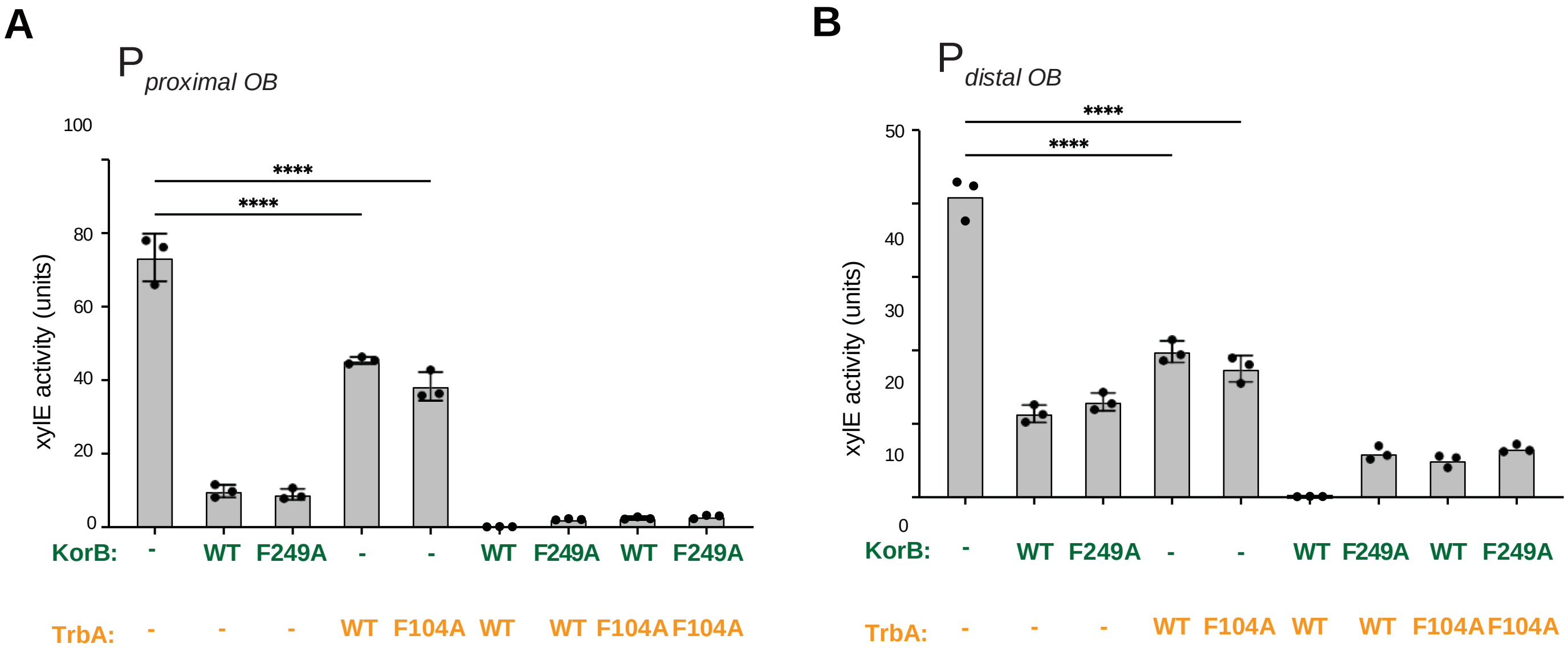

Supplementary Fig. 3. TrbA cooperates with KorB to repress transcription from a distance *in vivo*. Values shown are absolute values of XylE activities from the corresponding panel in Fig. 4B**.** Experiments were performed in triplicate and mean +/- SDs were presented. *P* values were calculated by ordinary one-way ANOVA; ****P $\leq$ 0.0001.
